# Reply to: Computer science work and interest profiles: stereotype vs. realities

**DOI:** 10.1038/s41598-023-47965-1

**Published:** 2023-12-11

**Authors:** Jenna McChesney, Tara Behrend, Alexander Glosenberg

**Affiliations:** 1 Meredith College, Raleigh, NC USA; 2 Michigan State University, East Lansing, MI USA; 3 Loyola Marymount University, Los Angeles, CA USA

**Keywords:** Computer science, Information technology

**replying to**: R. Su et al.; *Scientific Reports* 10.1038/s41598-023-47963-3 (2023).

## Introduction

We thank Su et al.^1^, herein our colleagues, for engaging with our study. In their Matters Arising article, they raise three primary concerns regarding our study, which found that the career interests of many current and aspiring computer scientists diverge from stereotypes of the field. Our colleagues argue that our definition of occupational interest profiles (OIPs), categorization of computer science (CS) jobs, and interpretation of heterogeneity in interests were incorrect or misleading. We disagree and make three observations. First, our definition of OIPs is supported by the literature and O*NET’s usage of them. Second, our colleagues appear to be using a conservative definition of CS that is not pertinent to the purpose of our original study; regardless, a re-analysis of our data using their definition does not alter our conclusions. Third, the key inference from our study holds regardless as to the level of heterogeneity of interests in other professions. We further observe that Su and colleagues problematically characterize data from O*NET in a way that appears to color it as an objective characterization of work when in reality any analyses of work involves multiple subjective inferences. While we agree that O*NET is the best available source for information about work, we call for continued research into ways in which it can be improved.

## The nature of OIPs

Our colleagues’ first concern is that we mischaracterized occupational interest profiles (OIPs) in two ways: first, by framing them as stereotypical in nature, and second, by characterizing them as “inferred interests of job incumbents.” Regarding the first claim, we did not intend to characterize OIPs as inherently stereotypical in nature—and we referenced the rigorous methodology underlying their construction in our measures section. Our use of the word “stereotypical” was in reference to societal descriptions of CS as non-social/-collaborative in nature^3^. Profiles in our data that resembled these stereotypes were labeled as stereotypical^2^. Nevertheless, we stand by our original claim that “it is possible… stereotypes are unwittingly reflected in O*NET’s estimations of the interests of those in CS professions”^2^. As support for this claim we note that O*NET ratings of CS-related occupations tend to be relatively low on social interests. The degree to which OIPs for other occupations match stereotypes of other fields is an empirical, and important, question.

Our colleagues appear to be concerned about public perceptions regarding OIPs and O*NET in general. We believe that data from O*NET represents our best source of information about work, something we emphasized in our original paper^2^. Moreover, we respect the work of our colleagues, who have made essential contributions to building and maintaining O*NET over the last two decades^4–6^. Nevertheless, ratings of O*NET interests involve the human judgment of both incumbent self-report and the judgment of human raters (see Rounds et al.^4–6^). We agree that O*NET is a rigorous effort to understand work, but to characterize its data as definitive and presumably free of subjectivity risks obscuring important limitations, underlying assumptions, and inferences inherent in the analysis of work^7^. In particular, they make the point that the tasks and activities OIPs are based on were “identified via a rigorous, systematic data collection process”^1^, yet neither its rigor nor systematic approach eliminates the potential for human subjectivity due to the unique characteristics of those sampled (and not sampled), the psychological tendencies inherent in the self-report of occupational incumbents, and the human judgment of the graduate students used in the construction of some O*NET ratings^5,6^.

Our colleagues also argue that we mischaracterized OIPs by describing them as “inferred interests of job incumbents”^1^. Our colleagues are correct that “an OIP was developed to reflect the extent to which Holland’s^2^ RIASEC work environments are descriptive and characteristic of core tasks and activities typically performed in an occupation”^1,4^. The point at issue for our colleagues appears to be whether OIPs focus on the typical characteristics of work environments versus the typical interests of incumbents. While it would have been more accurate for us to have stipulated this distinction, and the underlying theories of person-environment (P-E) fit, in greater depth, given space constraints and the focus of the paper on other matters, we judged that it was not necessary.

This decision is supported by the theoretical and practical blurring of the interests of job incumbents who thrive and persist in a job and the typical nature of the job environment. From a theoretical perspective, John Holland, the founder of the theory of P-E fit underlying the approach to interests utilized by O*NET, believed that “people in the environment are the environment”^4^. Such a belief is supported by a core assumption of P-E fit—that people seek out environments that allow them to manifest their interests, that a better fit between a person’s interests and their environment leads to positive outcomes like satisfaction and lower turnover, and that this process is ongoing and reciprocal^8^. As a result, the interests of the typical incumbents who thrive and persist in a work environment will tend to match the characteristics of that work environment, and vice versa. From an applied perspective we note that OIPs are not presented to the public as just characteristics of work environments. In prominent places on O*NET’s website, they are labeled simply as “interests” or “occupational interests” and interests are defined as “preferences for work environments and outcomes”^9,10^. Because preferences are held by people, not environments, O*NET itself functionally represents OIPs as the inferred interests of typical or well-fitting job incumbents—helping to support our use of a similar shorthand definition for OIPs in the original study.

## Jobs classified as computer science occupations

The second concern raised by our colleagues is that we incorrectly categorized occupations as relating to computer science (CS). In support of their claim, they reference occupational categories developed by the United States Department of Labor (DOL)—stating “computer science occupations should belong to SOC category 15-0000 Computer and Mathematical Occupations”^1^. We observe that the DOL’s standard occupational classification (SOC) system was developed for a variety of federal administrative uses, and it was originally devised using “practical approaches to classification rather than for (perhaps more appealing) theoretical approaches” (see Levine et al.^11^). In contrast, in line with our goal of exploring a generalist understanding of CS professions, we used conceptualizations of CS professions from individuals from an assortment of backgrounds. As our colleagues point out, this categorization led to a more inclusive definition of CS professions than those occupations in SOC 15-0000. We note that our demarcation included such professions as “intelligence analysts,” and “microsystems engineers,” professions that represent the forefront of how CS is revolutionizing today’s society—and occupations that would otherwise be excluded using our colleagues’ criteria. Moreover, our criteria included occupations like “robotics technicians,” which, according to O*NET^12^, require less education than many other CS occupations but still involve prototypical CS activities, such as programming complex systems.

Nevertheless, we re-analyzed our data (see SI Appendix) using our colleagues’ definition of CS professions, which led us to the same conclusion we reached in our original study. That is, a meaningful number of men and women employed in CS occupations hold interests that are not well-aligned with stereotypical depictions of CS professions as non-collaborative. Moreover, we again observed relatively high levels of artistic interests. Therefore, it is not the case, as our colleagues speculate, that our results are due to what they characterize as our misrepresentation of the CS professions and its members.

## Interest diversity within occupations

The third concern raised by our colleagues is a combination of the following: that data from O*NET can offer invaluable career guidance, that a diversity of interests within occupations are to be expected, and that we were misleading by not better highlighting such. We agree with both the first and second of these assertions but disagree with the third. As observed by our colleagues, variance in interests is likely to be expected across occupations^13^. However, regardless as to whether the variance observed herein is similar to, greater than, or lesser than the variance observed in other types of occupations, the key insight from our study still holds. This insight is that a meaningful number of people employed in CS, including women, appear to hold interests different than what would have been expected based upon stereotypical depictions of CS as non-collaborative and socially isolating. Because women have been traditionally underrepresented in CS occupations^14^, our conclusion is that at least some women might be dissuaded from participating in CS occupations if a greater diversity of interests in CS professions is not communicated to the public. While speculative, this is an important question for continued research. Moreover, due to their historical underrepresentation, even a small number of such women is meaningful. Thus, even using non-representative sampling, our study can help to justify continued research into interest variability within the CS profession, and on the effects of exposing individuals to different types of occupational-interest information on career development.

We thank our colleagues for helping us to clarify the above issues and heartily agree with them that relational job design^15^ is a promising path forward in the greater inclusion of women in CS professions. We hope that our reply underscores that any particular approach to analyzing the nature of work is not definitive and inevitably involves human subjectivity and the limitations of particular work-analytic approaches. It is our desire that this discussion leads to the continued improvement of O*NET—arguably the world’s best approach to understanding the world of work—by considering to what extent the perspectives and realities of underrepresented populations might not be optimally represented in its approach.

### Supplementary Information

Supplementary Information.
