# Supplementary material for: Reply to: Computer science work and interest profiles: stereotype vs. realities
Source: Sci Rep. 2023 Dec 11;13:21924. doi: 10.1038/s41598-023-47965-1 (PMC10713566; doi:10.1038/s41598-023-47965-1)
Supplement: Supplementary file 1 — Supplementary Information. [file 41598_2023_47965_MOESM1_ESM.docx]

Supporting Information

Reply to: Computer Science Work and Interest Profiles: Stereotype v. Realities

Jenna E. McChesney^1*^, Tara Behrend^2^ & Alexander Glosenberg^3^

^1^Meredith College, Raleigh, NC

^2^ Michigan State University, East Lansing, MI

^3^Loyola Marymount University, Los Angeles, CA

*Correspondence: [jemcchesney@meredith.edu](mailto:jemcchesney@meredith.edu)

As mentioned in our reply to Su and colleagues [1], we re-analyzed our data using the definition they advanced. Specifically, we removed responses from occupations that were not in the 15-000 Standard Occupational Classification (SOC) category.^[[1]](#footnote-1)^ The removal of jobs belonging to 11 other occupational categories (Table S1) reduced the available sample of aspiring computer scientists from 603 to 338 responses and the sample of employed computer scientists from 4,059 to 2,637 responses. Because a sample size of at least 500 is recommended to conduct LPA [2], we were unable to perform LPA on the re-categorized sample of aspiring computer scientists (*N*=338). However, a large enough sample was retained to re-analyze the employed data (*N*=2,637). The titles and occupational codes of the jobs characterized as CS in this sample are provided in Table S2. This table can be compared to Tables 2 and S3 in the published manuscript [3].

As in our original study and aligned with previous research [4], we estimated models ranging from two to ten profiles, preferring solutions that did not have classes with a small number of individuals. We used Akogul and Erisoglu’s [5] analytic hierarchy process (AHP) to select the model with the optimal number of profiles. The AHP takes a holistic approach when considering information criteria, such as Akaike’s Information Criterion (AIC) and Bayesian Information Criterion (BIC), to select the best model.

We found eight distinct profiles for those employed in the re-classified CS occupations (AIC = 52085.66, BIC = 52085.34). This is in comparison to 10 profiles from the full (N=4,059) sample (see Figure S3 in the original SI) and four from a randomized sub-sample (N=500) in the original manuscript (Figure 1). Descriptive labels and overall characterizations of the structures of those profiles are provided in Table S3 based upon Holland’s (1997) RIASEC taxonomy (see our original paper). We re-estimated O*NET CS RIASEC scores by using an average of the reclassified CS professions – an average weighted by the representation of each profession in our sample of 2,637 responses. We also re-include the average RIASEC scores for all 974 occupations with available information from O*NET (herein the U.S. occupational average) for comparison purposes. Profile scores, the overall O*NET CS estimate, and the U.S. occupational average are displayed in Figure S1.

Of the eight profiles found in the re-classified sample of employed computer scientists (N = 2,637), at least four of these profiles resembled those found in the original reported sample of employed computer scientists (i.e., Artistic, Multi-interested, Uninterested, and Stereotypical). The Artistic profile found in the original sample was visually similar to Profile 1 with relatively high interests in artistic tasks. It also had a relatively high correlation with, and low distance score comparison to, Profile 1 (*r* =.99; Dist. = 2.58). The Multi-Interested profile found in the original sample was visually similar to Profile 3 with high interests in all tasks. It also had a relatively high correlation and low distance score comparisons to Profile 3 (*r* = .98; Dist. = .70). The Uninterested profile found in the original sample visually resembled Profile 7 with relatively low interests in all tasks and relatively high correlation and low distance score comparisons (*r* = .93; Dist. = 2.71). Finally, the Stereotypical profile found in the original sample was visually similar to Profile 4 with high interests in realistic tasks and low interests in artistic tasks and with relatively high correlation and low distance score comparisons (*r* = .98; Dist. = 2.65).

Chi-square tests were used to determine whether significant gender differences existed across profiles. As in the original study, gender was significantly related to profile membership for those employed in the re-categorized CS-related occupations (χ2(7) = 252.55, p < 0.001). We also again noted relative higher representation of women (68%) among responses from Artistic profiles and lower representation in Stereotypical profiles (17%). Beyond just profiles that closely resembled profiles in our original study (see the original Figure S3 and Table S4), we again observed multiple profiles (namely, Profiles 2 and 6; see Figure S1 and Table S3 herein) where Social scores were relatively high – in particular, higher than the U.S. average, and higher than Realistic scores. This is the reverse pattern of the CS O*NET scores where Realistic scores are relatively higher than Social scores, and Social scores are below the U.S. average. Both of these profiles held a majority of women. In fact, 477 women from our sample belonged to one of these three profiles (Profiles 1, 2, or 6; see Table S3 herein). In contrast, as indicated in Table S3, multiple profiles (Profiles 5 and 8) identified in the new sample exhibited a relatively close correspondence with the O*NET estimate of CS interests in terms of correlations and distance scores. These profiles both held a majority of responses from men.

In conclusion, using the definition of CS from Su and colleagues (under review), we again conclude that the interests of many persons, including many women, in our sample do not closely match the interests reflected by O*NET’s CS weighted average.

| **SOC Code** | **Category Label** |
| --- | --- |
| 11-000 | Management Occupations |
| 13-000 | Business and Financial Operations Occupations |
| 17-000 | Architecture and Engineering Occupations |
| 19-000 | Life, Physical, and Social Science Occupations |
| 27-000 | Arts, Design, Entertainment, Sports, and Media Occupations |
| 33-000 | Protective Service Occupations |
| 39-000 | Personal Care and Service Occupations |
| 43-000 | Office and Administrative Support Occupations |
| 49-000 | Installation, Maintenance, and Repair Occupations |
| 51-000 | Production Occupations |
| 53-000 | Transportation and Material Moving Occupations |

**Table S1.** The 11 SOC Categories “Incorrectly” Classified as CS in the Original Study According to Su et al.

| **Code** | **Title** | **# of responses** |
| --- | --- | --- |
| 15-1132.00 | Software Developers, Applications | 380 |
| 15-1199.09 | Information Technology Project Managers | 377 |
| 15-1121.00 | Computer Systems Analysts | 252 |
| 15-2031.00 | Operations Research Analysts | 211 |
| 15-1151.00 | Computer User Support Specialists | 187 |
| 15-1134.00 | Web Developers | 143 |
| 15-1133.00 | Software Developers, Systems Software | 132 |
| 15-1199.08 | Business Intelligence Analysts | 129 |
| 15-1142.00 | Network and Computer Systems Administrators | 127 |
| 15-1131.00 | Computer Programmers | 112 |
| 15-1199.10 | Search Marketing Strategists | 99 |
| 15-1122.00 | Information Security Analysts | 70 |
| 15-1111.00 | Computer and Information Research Scientists | 65 |
| 15-1199.01 | Software Quality Assurance Engineers and Testers | 54 |
| 15-1141.00 | Database Administrators | 53 |
| 15-1143.00 | Computer Network Architects | 53 |
| 15-1199.02 | Computer Systems Engineers/Architects | 49 |
| 15-1199.06 | Database Architects | 47 |
| 15-1152.00 | Computer Network Support Specialists | 39 |
| 25-1021.00 | Computer Science Teachers, Postsecondary | 17 |
| 15-1199.05 | Geographic Information Systems Technicians | 12 |
| 15-1199.03 | Web Administrators | 11 |
| 15-1199.07 | Data Warehousing Specialists | 7 |
| 15-2041.02 | Clinical Data Managers | 7 |
| 15-1199.11 | Video Game Designers | 4 |

**Table S2.** Occupations classified as CS by our responding colleagues and the number of responses from our data of persons identifying as being employed in those occupations

|  | Responses | | |  | Average Interest Dimension Score | | | | | |  | O*NET Comparison | |
| --- | --- | --- | --- | --- | --- | --- | --- | --- | --- | --- | --- | --- | --- |
| Profile | Total  (N=2,637) | Women  (N=1,141) | |  | R | I | A | S | E | C |  | D | r |
| Profile 1/Artistic (68% women) | 187 | | 127 |  | 2.05 | 4.24 | 6.33 | 4.09 | 3.83 | 2.81 |  | 11.43 | -.43 |
| Profile 2  (67% women) | 126 | | 85 |  | 1.79 | 2.33 | 2.34 | 3.91 | 4.56 | 3.35 |  | 9.63 | -.21 |
| Profile 3/Multi-Interested (38% women) | 392 | | 148 |  | 4.70 | 4.57 | 5.71 | 3.83 | 3.79 | 4.11 |  | 8.05 | -.16 |
| Profile 4/ Stereotypical (17% women) | 314 | | 52 |  | 4.83 | 3.63 | 1.76 | 2.75 | 3.60 | 4.33 |  | 5.15 | .65 |
| Profile 5  (45% women) | 225 | | 102 |  | 2.32 | 4.59 | 1.71 | 2.77 | 3.49 | 4.05 |  | 5.09 | .82 |
| Profile 6 (57% women) | 461 | | 265 |  | 2.01 | 3.97 | 4.29 | 3.30 | 3.26 | 3.01 |  | 9.14 | -.10 |
| Profile 7/Uninterested (49% women) | 432 | | 212 |  | 1.60 | 2.08 | 1.33 | 2.06 | 2.52 | 2.47 |  | 10.55 | .45 |
| Profile 8/  (30% women) | 500 | | 150 |  | 4.31 | 4.11 | 3.59 | 3.39 | 3.86 | 4.16 |  | 5.56 | .83 |
| O*NET CS Interests | - | |  |  | 3.79 | 5.56 | 2.34 | 1.89 | 3.76 | 4.91 |  | - | - |
| U.S. occupational average | - | |  |  | 4.73 | 3.45 | 2.17 | 2.84 | 3.49 | 4.16 |  | - | - |

**Table S3.** Interest Profile Scores of Employed Computer Scientists (2,637 responses) Compared to O*NET Estimates of CS Interests (R=Realistic, I=Investigative, A=Artistic, S=Social, E=Enterprising, C=Conventional, D=distance scores; *r*=correlations). Interests were assessed in both the online career interest survey and O*NET according to a scale from 1 to 7 with 7 indicating a stronger preference for / a greater relevance of that interest to the occupation.


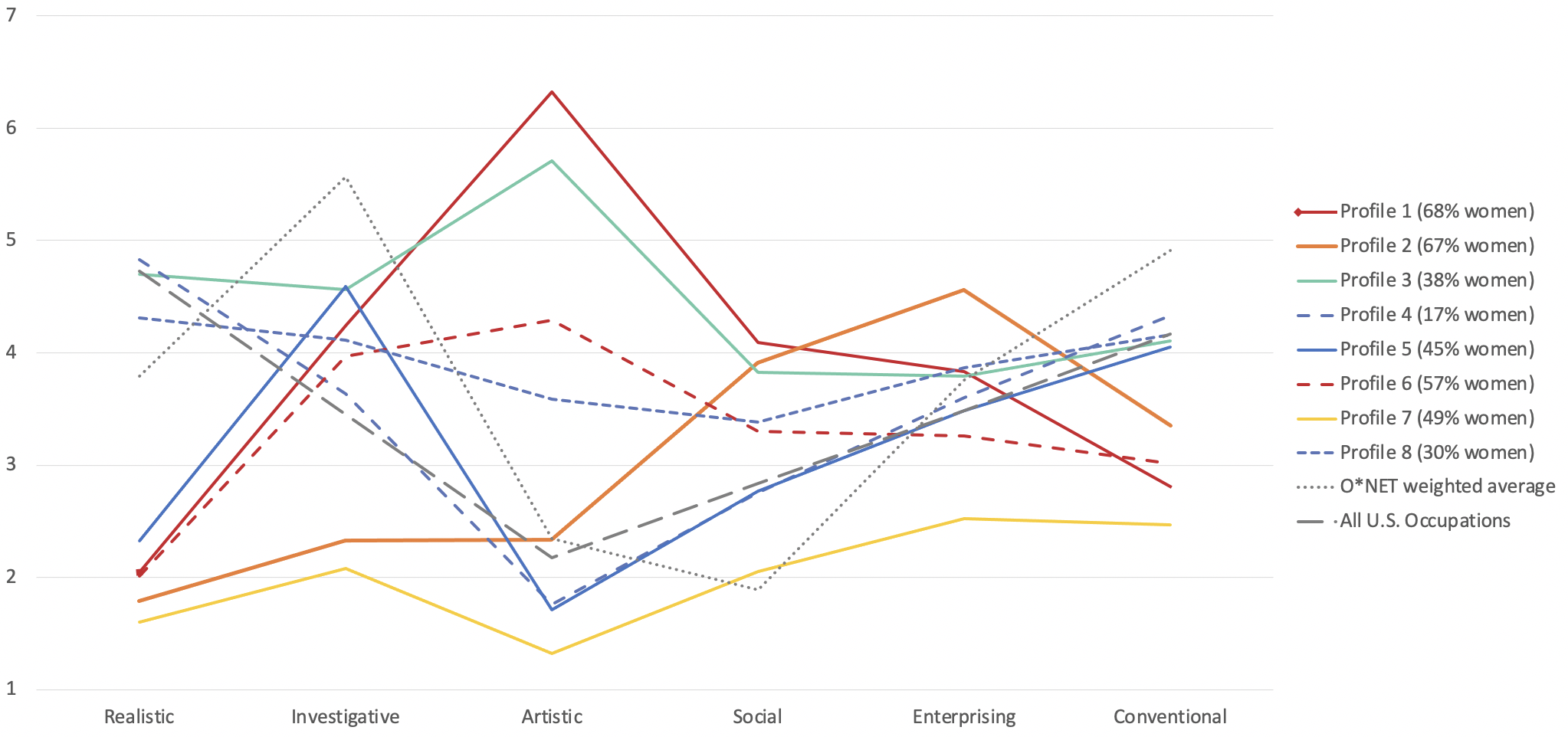


**Figure S1.** Interest Profiles of Those Employed in the Re-classified CS Occupations Compared to O*NET Estimations of CS Interests. Interests were assessed in both the online career interest survey and O*NET according to a scale from 1 to 7, with 7 indicating a stronger preference for/a greater relevance of that interest to the occupation.

**Method**

As in our original study, our characterization of CS interests was drawn from responses to an online survey posted to Time Magazine’s website (see www.time.com; herein the career interest survey). These data were collected in June 2016 and are available from the Open Science Framework (https://bit.ly/3nCBOUN). The study's first author secured institutional review board (IRB) approval (IRB protocol #14112) to utilize archival data derived from this survey. The survey was promoted on the magazine's website as a means for readers to explore their career interests and assess their alignment with occupational aspirations or characteristics. The survey link, accessible as of June 2023, can be found at <https://time.com/4343767/job-personality-work/>.

In the survey, participants were requested to provide self-reported demographic information, including age, gender, educational attainment, employment status, and personal income. Those currently employed were asked to specify their current job, while those who identified as unemployed were asked to indicate their aspirational ("dream") job. Additionally, respondents were required to answer questions regarding their interests in twenty various work activities. Upon completing the survey, participants were presented with a visual comparison between their interest levels in different tasks and the typical characteristics associated with their current or dream job.

**Matching of Respondents from the Career Interest Survey to Occupations**

Occupational titles associated with respondents' current or dream jobs were determined through a dynamic keyword search. This search process involved matching the job titles provided by respondents in everyday language (e.g., teacher, farmer) with the corresponding occupational titles used in O*NET's classification system (e.g., elementary school teachers, farmworkers).

**Categorization of CS Occupations**

Computer science (CS) occupations were categorized according to Su and colleagues’ definition. Occupations belonging to SOC 15-0000 Computer and Mathematical Occupations were categorized as CS. Only one occupation, 25-1021.00 (Computer Science Teachers, Postsecondary), was included outside of the 15-0000 category because Su and colleagues (under review) did not include it in their list of misclassified occupations. The titles and occupational codes of the jobs classified as CS are presented in Table S2. For further reference, this table can be compared to Tables 2 and S3 in our original published manuscript.

**Sample**

The online survey posted to Time Magazine’s website generated 84,394 responses and we isolated the 40,646 responses to the survey that were provided by those who were 18 years or older and whose internet protocol addresses were from the United States. Consistent with the original manuscript, we eliminated non-U.S. responses. We further isolated 2,975 responses from individuals employed or interested in the occupations that had been categorized as CS using the procedure explained above. After eliminating jobs from 11 occupational categories (see Table S1), the initial pool of aspiring computer scientists decreased from 603 to 338 responses, while the sample of employed computer scientists decreased from 4,059 to 2,637 responses. Due to the recommended minimum sample size of 500 for conducting Latent Profile Analysis (LPA) [2], it was not feasible to perform LPA on the re-categorized group of aspiring computer scientists (N=338). Nevertheless, the retained sample size of 2,637 employed individuals was still sufficient for re-analyzing the data.

**Employed in CS**

As described above, 2,637 responses were categorized as “employed in CS”. Of these 2,637 responses, 57% were from men and 43% were from women. Demographics were similar to those reported in the original study. Most responses came from those with an undergraduate (49%) or postgraduate (35%) degree. Only 13% of responses were from individuals between the ages of 18-25 years old, 23% between 26-33 years old, 32% between 34-45 years old, 19% between 46-55 years old, 11% between 56-65 years old, and only 2% were 66 years old or older.

**Measures**

We used the same measures from the online career-interest survey that was used in the original published manuscript. Please see McChesney and colleagues [3] for more information about these measures.

**References**

1. Su, R., Putka, D., & Rounds, J. Computer science work and interest profiles: Stereotypes vs. realities. *Matters Arising Sci Rep* (under review).
2. Spurk, D., Hirschi, A. Wang, M., Valero,D.. & Kauffeld, S. Latent profile analysis: a review and “how to”guide of its application within vocational behavior research. *Journal of Vocational Behavior*. **120**, 103445 (2020).
3. McChesney, J. E., Behrend, T. S. & Glosenberg, A. Stereotypical descriptions of computer science career interests are not representative of many computer scientists. *Sci Rep* **12**, 5990 (2022).
4. H. N. Perera, & P. Mcllveen, Vocational interest proles: Prole replicability and relations with the STEM major choice and the Big-Five. Journal of Vocational Behavior, **106**, 84-100 (2018).
5. S. Akogul, & M. Erisoglu, An approach for determining the number of clusters in a model-based cluster analysis. Entropy. **19**, 9, 452 (2017).

1. We note that we included one occupation, 25-1021.00 (Computer Science Teachers, Postsecondary), outside of the 15-0000 category because Su and colleagues (under review) did not include it in their list of misclassified occupations. [↑](#footnote-ref-1)
